# Procoagulant Disorders in Patients with Newly Diagnosed Pancreatic Adenocarcinoma

**DOI:** 10.3390/medicina56120677

**Published:** 2020-12-09

**Authors:** Renata Talar-Wojnarowska, Małgorzata Woźniak, Anna Borkowska, Katarzyna Cypryk, Marek Olakowski, Ewa Małecka-Panas

**Affiliations:** 1 Department of Digestive Tract Diseases, Medical University of Lodz, Kopcinskiego 22, 90-153 Lodz, Poland; mmw89@op.pl (M.W.); ewa.malecka-panas@umed.lodz.pl (E.M.-P.); 2 Department of Internal Diseases and Diabetology, Medical University of Lodz, Pomorska 251, 92-213 Lodz, Poland; anna.borkowska@umed.lodz.pl (A.B.); katarzyna.cypryk@umed.lodz.pl (K.C.); 3 Department of Digestive Tract Surgery, Silesian Medical University, Medyków 14, 40-772 Katowice, Poland; olakom@mp.pl

**Keywords:** pancreatic adenocarcinoma, coagulopathy, tissue factor, thrombin-antithrombin complex, sP-selectin

## Abstract

*Background and objectives*: Cancer coagulopathy is thought to be partially due to the up-regulation of tissue factor (TF), thrombin-antithrombin complex (TAT) and soluble P-selectin (sP-selectin). The purpose of this study was to evaluate the clinical significance of TF, TAT and sP-selectin in patients with pancreatic cancer. *Materials and methods:* The study included 93 subjects: 73 newly diagnosed patients with pancreatic adenocarcinoma (42 with stage I-III and 31 with metastatic cancer (stage IV)) and a control group of 20 healthy subjects. Analyzed patients were hospitalized in the Department of Digestive Tract Diseases, Medical University of Lodz or in the Department of Digestive Tract Surgery, Silesian University, Katowice, Poland. All laboratory parameters were measured using ELISA procedures. *Results:* TF plasma levels were detectable in all patients and were significantly higher in metastatic cancer compared to stage I-III patients and the control group (*p* < 0.05). In patients with pancreatic adenocarcinoma, the median levels of TAT were also elevated compared to the control group. Moreover, patients with metastases had significantly higher TAT concentration compared to the I-III cancer group. On the other hand, only the metastatic patients group showed significantly higher plasma sP-selectin levels compared to the controls (*p* = 0.009), whereas there was no difference between localized and metastatic cancer patients. *Conclusions:* The coagulation disorders are present in the majority of patients with pancreatic adenocarcinoma already at the diagnosis stage and reflect cancer progression and spread.

## 1. Introduction

Pancreatic adenocarcinoma (PA) is commonly associated with thrombotic events, which contribute to its morbidity and mortality [1,2,3,4]. Venous thromboembolism (VTE) occurs in over one-third of PA patients and is the second leading cause of their death [5]. A thrombophilic environment frequently arises in cancer and involves the capacity of tumor to express and release clotting factors, inflammatory cytokines, proangiogenic and procoagulant factors. One obvious manifestation of cancer-associated coagulopathy is the increased risk of VTE among cancer patients; however, the procoagulant state may also promote tumor progression [3,6].

Cancer coagulopathy is thought to be partially due to the up-regulation of tissue factor (TF), a 47-kd glycoprotein expressed on the cell surface, which is the principal physiologic initiator of the extrinsic coagulation pathway as factor VII/VIIa receptor. Recently, TF has gained considerable attention as the determinant of tumor progression, not only by its proteolytic activity via the coagulation cascade, but also by its intracellular signalling [7]. TF is commonly expressed in a variety of cancers and has been correlated with stage and outcome in a variety of solid tumors, including easophegal, breast and ovarian cancers [8,9,10]. In pancreatic cancer, high-grade TF expression was reported as the negative prognostic factor [11]. It has been proved that TF expression occurs early in pancreatic neoplastic transformation and is associated with vascular endothelial growth factor (VEGF) expression, microvessel density, and possibly clinical VTE in pancreatic cancer [12,13]. In our previous study, we also demonstrated increased VEGF levels associated with larger tumor size, lymph node involvement and the presence of liver metastases in PA patients [14].

It is also known from the literature that, in oncological patients, increased levels of thrombin-antithrombin complex (TAT) may be observed, which reflects the activation of thrombin levels and coagulation [15,16,17,18]. TAT levels in plasma were significantly elevated in patients with breast cancer and were associated with increased CA15-3 tumor marker [16]. Similarly, among ovarian cancer patients, TAT concentrations were correlated with advanced tumor stage and CA125 levels [17]. The data also confirmed the occurrence of an increase in TAT levels and the activation of hemostasis in patients with pancreatic cancer. In a recently published study, PA patients with VTE had significantly higher TAT levels, strongly associated with VTE risk even in asymptomatic patients [18]. However—to our best knowledge—there is no more comparable studies about TAT levels in PA patients.

Accumulating data suggest that P-selectin, a member of the selectin family of cell adhesion molecules, might also play an important role in the interrelation between cancer and thrombosis [19]. Moreover, cancer cells are able to enhance P-selectin expression on monocytes, macrophages, endothelial cells, and platelets. It is known that neoplastic cells express on their surface the ligand CD24, which was identified to be a receptor for P-selectin. The interaction of P-selectin with CD24 on neoplastic cancer cells allows for their interaction with platelets and their adherence to endothelium in the process of metastatic spread [20]. P-selectin is present in blood as soluble form—sP-selectin—and increases in inflammation and carcinogenesis. Some studies have demonstrated that the high plasma levels of soluble P-selectin are strongly associated with VTE and cancer prognosis [21,22]. On the other hand, Graf et al. have recently proved in a large prospective study that pro-coagulative factors, including sP-selectin, are probably not related to the increased risk of common cancers; however, enhanced platelet activation may drive cancer progression and metastases [23].

Due to ambiguous data from literature about the role of procoagulant factors in pancreatic carcinogenesis, this issue needs further evaluation. The purpose of this study was to evaluate the clinical significance of TF, TAT and sP-selectin in patients with PA, before any anticancer treatment.

## 2. Materials and Methods

The study included 93 patients: 73 with newly diagnosed pancreatic cancer and 20 healthy volunteers as the control group. PA patients included 42 diagnosed with stage I-III (25men and 17 women aged 53–81) and 31 patients with metastatic PA (stage IV) at the time of diagnosis (18 men and 13 women aged 51–87). A control group consisted of 20 gender- and age-matched healthy subjects who had no evidence of neoplastic history and acute or chronic diseases. Analyzed patients were hospitalized in the Department of Digestive Tract Diseases, Medical University of Lodz or in the Department of Digestive Tract Surgery, Silesian University, Katowice, Poland, in 2018–2019. Written informed consent was obtained for all the patients. The study protocol was approved by the ethical committee of the Medical University of Lodz. (nr RNN/30/18/KE, 12. 06. 2018.)

The pathologic diagnosis was confirmed in all subjects after surgical treatment or after EUS-guided pancreatic tumor biopsy in patients qualified for palliative chemotherapy. Patients were divided into two groups, according to the tumor-node-metastasis (TNM) classification, a wordwide benchmark for reporting the extent of malignant disease. Patients were stratified according to the presence of distant metastases; the first group included patients without metastases (any T, any N, M0; stage I-III) and the second group included metastatic PA patients (any T, any N, M1; stage IV). The diagnosis of metastatic PA was based on the computed tomography (CT) or magnetic resonance of the abdomen cavity and chest x-ray or CT exam. Patients with a history of thromboembolism before PA diagnosis and administered anticoagulant or antiplatelet drugs were excluded from this study. The associations of the TF, TAT and sP-selectin and clinical data of PA patients at diagnosis have been evaluated, before any anticancer treatment. Moreover, we investigated the correlation between analyzed parameters and routinely assessed laboratory data, including D-dimers, C-reactive proteins (CRP), platelets (PLT) and haemoglobin (HGB) levels.

The peripheral venous blood samples were collected from each patients at the time of the clinical diagnosis of pancreatic cancer after signing a consent form. The blood was drawn into sterile vacuum tubes with ethylenediaminetetraacetic acid (EDTA), separated by centrifugation at 3000 revolutions per minute within 30 min of collection and stored at −20 °C until the levels of analyzed markers were assessed.

The sP-selectin and TF levels in the plasma were measured using specific enzyme-linked immunosorbent assay (ELISA) R&D Systems in accordance with the test procedure. The sensitivity assays were evaluated and the minimum detectable dose of human TF ranged from 0.16–2.05 pg/mL and dose of sP-selectin was 0.5 ng/mL. There was no cross reactivity with other plasma proteins. The TAT plasma concentrations were measured with ELISA according to the producer recommendations (Wuhan Fine Biotech Co., Fine Test). Results were expressed in pg/mL. No significant cross-reactivity or interference between TATC and analogues was observed. The hemolyzed samples were unsuitable for use in all those tests. In addition, samples were analyzed for routine peripheral blood cells (e.g., hemoglobin and platelets) as well as D-dimers and CRP, prior to any therapeutic procedure, including surgery.

### Statistical Analysis

Measured values were expressed as mean ± SE. Significance of differences between studied groups was calculated by t-Student test, Mann–Whitney U test and χ^2^ test. P-values less than 0.05 were considered statistically significant. All statistical calculations were performed using Statistica 13 for Windows (Statsoft, Poland).

## 3. Results

Mean ages were not significantly different in patients with localized pancreatic cancer (mean 66.1 ± 4.1) and metastatic PA (69.2 ± 4.3; *p* > 0.05). The tumor size ranged from 1.5 to 5.1 cm in the first group of patients, which was significantly smaller than the tumor size of metastatic PA patients (2.3 to 6.5 cm respectively; *p* < 0.05). For histological differentiation 14, 13 and 16 patients of I-III group were classified into G1, G2 and G3, respectively, whereas, in metastatic patients, low-differentiated tumors were the most frequently observed (Table 1). 

The liver was the most common site of metastases (23 patients; 74.3%), followed by the lungs (nine patients; 29.3%), bones (three patients; 9.7%) and adrenal gland (one patient; 3.2%). Seven patients (22.6%) had distance metastases in liver and in lungs at the time of diagnosis, two patients (6.4%) in liver and bones and one patient (3.2%) had metastatic spread into the liver, lungs and kidney. Lymph node metastases were observed in 47 PA patients, more frequent in subjects with distant metastases (*p* < 0.001). We have not observed statistically significant difference between routinely analyzed laboratory parameters, including HGB, PLT, CRP and D-dimers, in local and metastatic PA patients. The descriptive characteristics of our study participants are displayed in Table 1.

**Figure 1:**
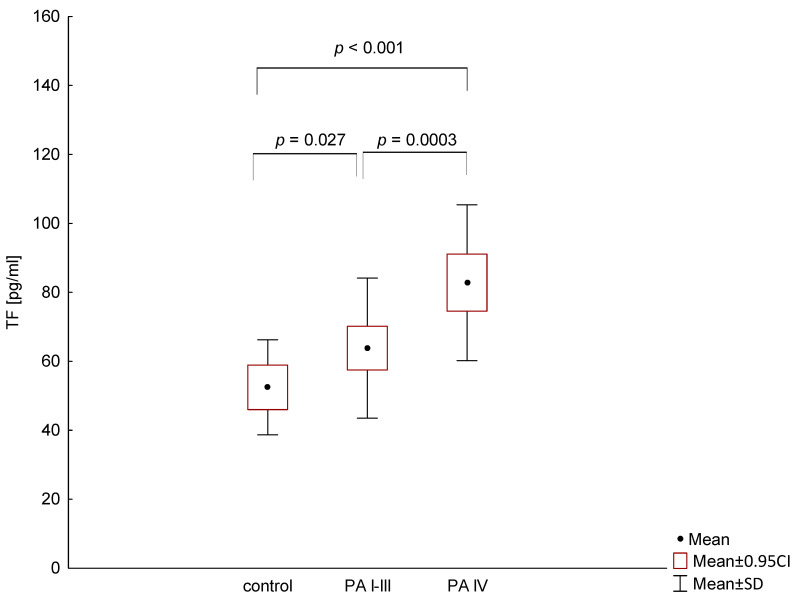
Comparison of tissue factor (TF) levels in patients with different stage of pancreatic adenocarcinoma (PA) and the control group.

TF plasma levels were detectable in all patients and were significantly higher in metastatic PA (mean cytokine level: 82.8 pg/mL; range 45.6–141 pg/mL) compared to stage I-III patients (63.8 pg/mL; range 32.7–112 pg/mL; *p* = 0.0003) and the control group (52.4 pg/mL; range 28.4–72.4; *p* < 0.001). There was also a significant difference in TF serum levels between healthy volunteers and patients with stage I-III (*p* = 0.027; Figure 1). 

**Figure 2:**
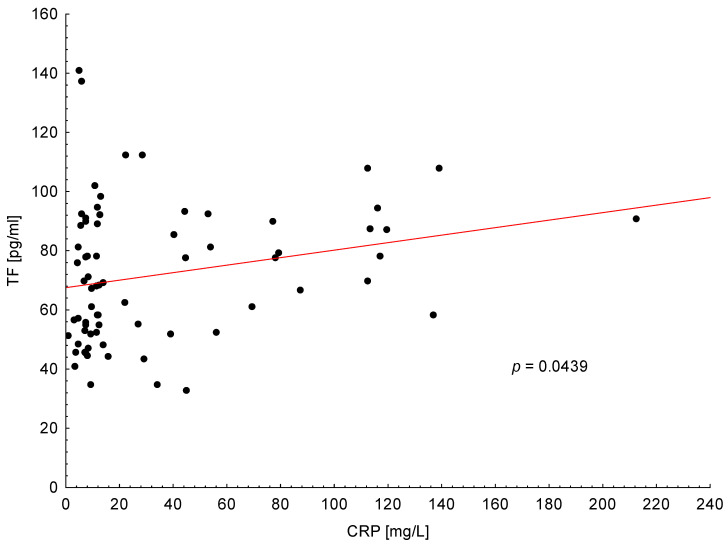
Positive correlation between tissue factor (TF) plasma levels and C-reactive protein (CRP) in all patients with pancreatic adenocarcinoma.

As shown in Figure 2, the positive correlation was found between TF plasma level and CRP in PA patients (*p* = 0.0439). There was not any other association between TF and clinical or laboratory features in analyzed group of patients.

**Figure 3:**
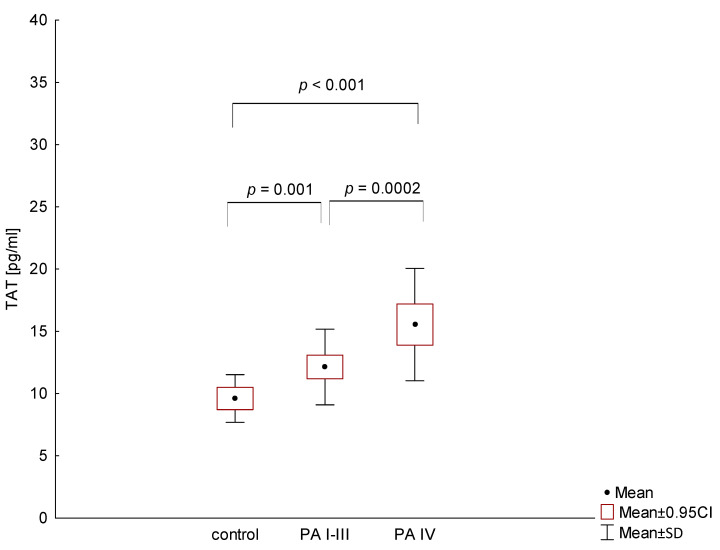
Comparison of thrombin-antithrombin complex (TAT) levels in patients with different stages of pancreatic adenocarcinoma (PA) and the control group.

In PA patients, the median levels of TAT were also elevated compared to the control group. Moreover, patients with metastases had a significantly higher TAT concentration compared to the I-III PA group (15.5 pg/mL versus 12.1 pg/mL, respectively; *p* = 0.002; Figure 3). 

**Figure 4:**
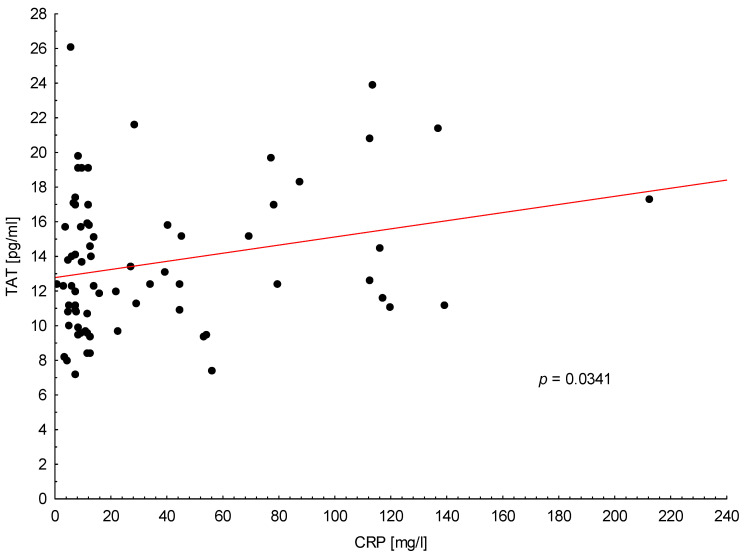
Positive correlation between trombin-antithrombin complex (TAT) plasma levels and C-reactive protein (CRP) in all patients with pancreatic adenocarcinoma.

Similarly, as with TF, TAT plasma level was associated with increased CRP level in all PA patients (*p* = 0.034; Figure 4). Furthermore, in metastatic PA patients, we observed the correlation between increased TAT levels and D-dimers (*p* = 0.041). In our study, plasma TAT concentration was not significantly associated with any other clinical or laboratory parameters.

**Figure 5:**
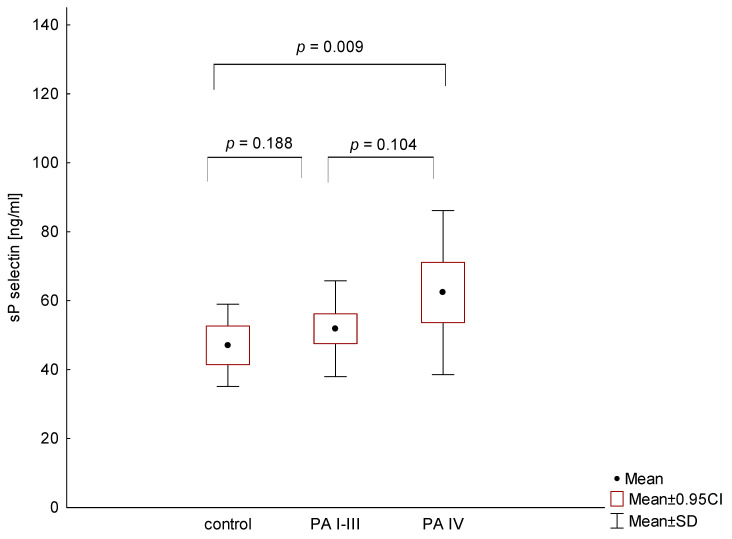
Comparison of sP-selectin levels in patients with different stages of pancreatic adenocarcinoma (PA) and the control group.

On the other hand, statistical analysis demonstrated that only metastatic PA patients showed significantly higher plasma sP-selectin levels compared to normal controls (*p* = 0.009), whereas there was no difference between localized and metastatic PA patients (*p* = 0.104; Figure 5). 

We also evaluated the relationship between sP-selectin levels and other parameters and found relevant correlation between sP-selectin and CRP in all PA patients (*p* = 0.0039, Figure S1). In addition, in metastatic PA patients, we observed a statistically significant negative correlation between sP-selectin and PLT levels (*p* = 0.037, Figure S2; shown in additional data).

## 4. Discussion

Pancreatic adenocarcinoma is a systemic disease, the majority of patients in the early stages of the cancer develop metastases and experience a dismal prognosis. Moreover, about 30–50% of PA patients present with an advanced stage of the disease or have distant metastases at the time of diagnosis [24]. As pancreatic carcinoma has a high propensity for both local invasion and distant metastases, surgical treatment is precluded for most patients who present with an advanced stage of the disease. Similarly, in our study, 42.5% of patients had metastatic disease at the diagnosis and were only qualified for palliative treatment.

Recent studies have shown the possible association of procoagulant factors and pancreatic cancer spread and prognosis [3,18,25]. Thrombosis and cancer are linked by numerous pathophysiological mechanisms that are generally related to the host response to cancer. These mechanisms include activation of the coagulation and fibrinolytic systems, acute phase reaction, inflammation, and cytokine production [3]. In the current work, we focused on assessing the coagulation factor disorders in PA patients. The advantage of this study is the homogenous cancer population, comprising only newly diagnosed PA patients, before any anticancer treatment. 

According to our data, plasma levels of TF were significantly higher in metastatic PA patients compared with other groups, a finding that may be caused by an excess production in tumor cells and a subsequent release into the circulation. Our results are in agreement with previously published studies which analyzed TF plasma concentrations in patients with other cancers, including pancreatic adenocarcinoma [10,13]. We proved that TF is a useful biomarker for identifying patients with metastatic PA, so may have a prognostic significance in clinical practice. It was prouved that the concentration of plasma TF was associated with its tissue expression in both tumor and stroma and with worse prognosis of oncological patients. TF overexpression was observed in the intestinal type of gastric cancer and it was associated with the advanced stage of the disease. Furthermore, it showed a positive correlation with a higher rate of lymphatic metastases and worse patient outcome [26]. Similarly, the multivariate analysis demonstrated that tumour TF expression was an independent prognostic indicator for overall survival in breast cancer patients [10]. On the other hand, in the study of Chen et al. TF immunoreactivity was significantly correlated to the presence of distant metastases in patients with esophageal cancer, while it was not correlated to patient’s gender, age, tumor size, depth of tumor invasion or lymph node involvement. However, the overall survival rate in the patients with high TF immunoreactivity was significantly poorer than those with low TF immunoreactivity [8].

In the current study, we also observed the increased plasma levels of TAT in PA patients, similar to other authors’ reports on patients with breast, ovarian and endometrial cancers [16,17]. However, we demonstrated that elevated TAT levels were present not only in metastatic PA patients but in majority of patients with pancreatic cancer at the time of diagnosis. The findings reported here are in agreement with recently published study of Mandoj et al., which found increased TAT plasma levels in 34.4% of women with early breast cancer, before any anticancer therapy [27].

We also observed the correlation between TAT levels and D-dimers; however, this was only in metastatic PA patients. There was no relationship between TAT levels and D-dimers in earlier stages of PA. Our reports are consistent with the data from the literature, suggesting that procoagulant factors are especially important in advanced neoplastic diseases with increased VTE risk. Moreover, Kondo et al. reported that TAT plasma levels as well as prothrombin fragment 1+2 and D-dimers were strongly associated with VTE risk in chemotherapy-naive PA patients. Furthermore, they observed that PA patients with VTE had significantly higher TAT levels that those without VTE. The TAT analysis may be useful for the early detection of VTE even in asymptomatic patients with PA [18]. The data suggest that TAT assay deserves further investigation; however, systematic studies are required to confirm the significance of these findings.

Another important aspect of our analysis was to assess the potential association of sP-selectin and clinical and laboratory features of PA patients. We demonstrated the relationship between elevated sP-selectin value and the presence of distal metastases in PA patients. Our results are in agreement with other studies which reported that higher plasma levels of sP-selectin were associated with the advanced stage of neoplastic diseases [28,29]. In patients with colorectal cancer, increased sP-selectin levels showed correlation with higher clinical grades of cancer, including lymph node involvement [29]. Interestingly, in several experimental studies, sP-selectin was considered a target for pharmacologic control of thrombosis as well as the metastasis process [30]. Ludwig et al. provided evidence that endothelial P-selectin expression may contribute to the formation of hematogenous metastases because melanoma cells might directly interact with post capillary venules in a P-selectin-dependent manner [31]. 

On the other hand, we did not observe the increased sP-selectin level in PA patients without metastases compared to the control group. The data from literatures about its clinical significance in neoplastic diseases are not uniform. In some studies, increased sP-selectin levels were also observed in earlier tumor stage; however, the data may be difficult to compare because of the different kinds of neoplastic tissues, so this issue needs further evaluation. However, it is known from the literature that high plasma levels of sP-selectin may represent a predictive parameter for the development of VTE in different site cancer patients. Ay et al. hypothesized that measurement of sP-selectin at the diagnosis of cancer would help to identify patients at an increased risk of VTE. However, well-designed, long-term prospective studies are needed to confirm the benefit from the prophylactic anticoagulant treatment of cancer patients with high levels of sP-selectin [32].

It is known that each neoplastic process is accompanied by intensified granulocytes and macrophages migration to the tumor stroma which causes leukocyte infiltration in pericancer tissue. The increased CRP level is often found in patients with neoplastic diseases, including PA [33,34,35]. CRP is directly associated with tumor mass and is believed to be induced by infiltrating lymphocytes and monocytes producing interleukins. In our study, elevated CRP was correlated with increased levels of all procoagulant factors in PA patients. Similar findings were reported by Kanz et al., who observed that increased CRP level was associated with VTE episodes and a worse prognosis of cancer patients [36].

## 5. Conclusions

In summary, we confirmed that the coagulation disorders are present in the majority of PA patients at the time of diagnosis and reflect cancer progression and spread. Importantly, our data demonstrate that TF and TAT are useful biomarkers for identifying patients with metastatic disease and have a prognostic potential. Our results are consistent with the biological function of those procoagulant factors and support the need for further research into their role in pancreatic adenocarcinoma.

## Figures and Tables

**Table 1:** Comparison of clinical and laboratory parameters between groups. (NS—not statistical).

	Group of PA Patients		*p*
	Stage I-III	Stage IV	
	*n* = 42	*n* = 31	
Mean age (years)	66.1 ± 4.1	69.2 ± 4.3	NS
Sex			
Male	25	18	NS
Female	17	13	
Tumor size			
<3 cm	24	9	<0.05
≥3 cm	19	22	
Tumor location			
Head of pancreas	32	23	NS
Body or tail	10	8	
Histological differentiation			
G1 + G2	27	14	NS
G3	16	17	
Lymph node involvement			<0.001
positive	18	29	
negative	25	2	
Hemoglobin (g/dL)	10.3 ± 1.3	9.6 ± 1.5	NS
Platelet (G/L)	251.2 ± 7.9	253.9 ± 9.1	NS
CRP (mg/L)	25.0 ± 2.7	46.7 ± 4.1	NS
D-dimers (ng/mL)	2162.2 ± 17.9	3475.5 ± 21.9	NS

## Supplementary Materials

The following are available online at https://www.mdpi.com/1010-660X/56/12/677/s1. Figure S1: Positive correlation between sP-selectin plasma levels and C-reactive protein (CRP) in all patients with pancreatic adenocarcinoma. Figure S2: Negative correlation between sP-selectin plasma levels and PLT in patients with metastatic pancreatic adenocarcinoma (stage IV).
