# Supplementary material for: Procoagulant Disorders in Patients with Newly Diagnosed Pancreatic Adenocarcinoma
Source: Medicina (Kaunas). 2020 Dec 9;56(12):677. doi: 10.3390/medicina56120677 (PMC7763230; doi:10.3390/medicina56120677)

Figure S1. Positive correlation between sP-selectin plasma levels and C-reactive protein (CRP) in all patients with pancreatic adenocarcinoma.

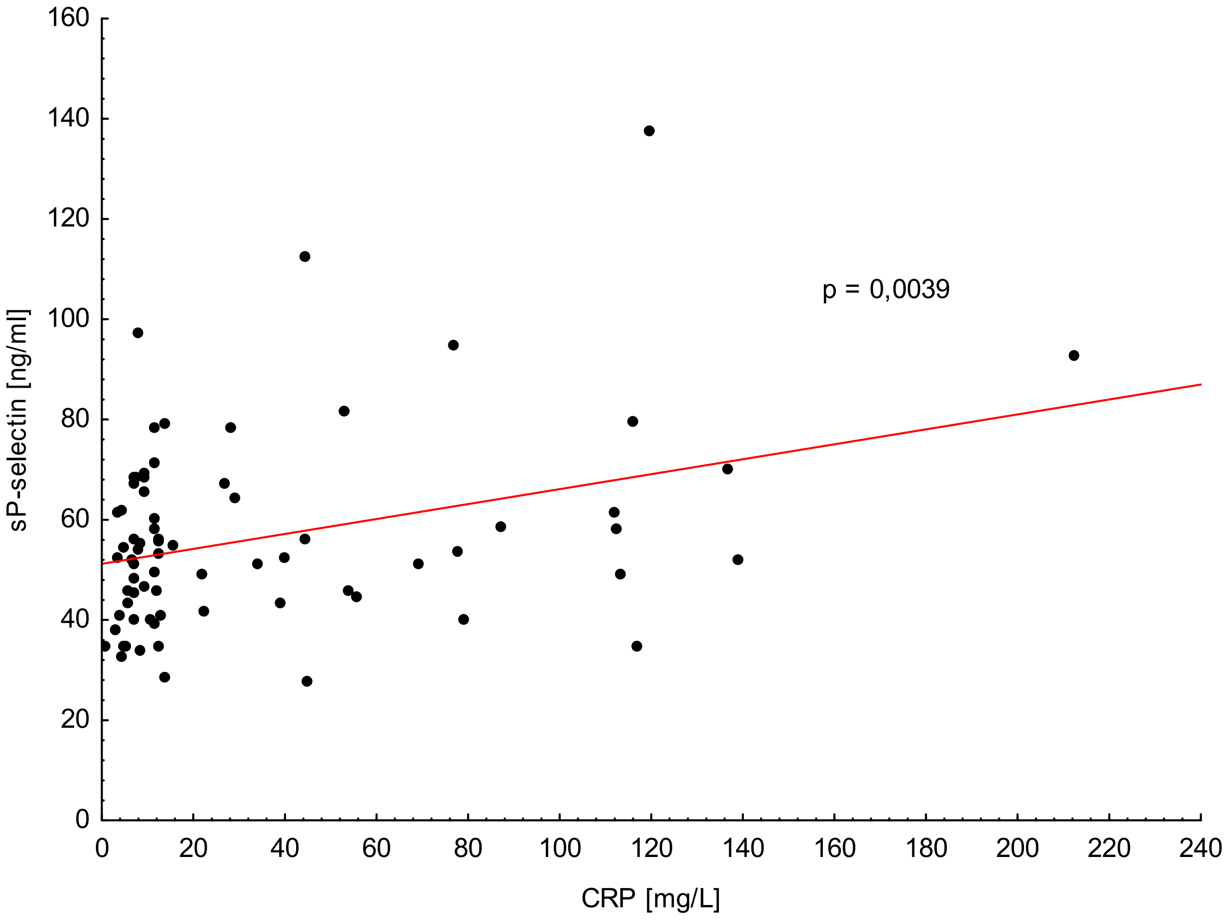

Figure S2. Negative correlation between sP-selectin plasma levels and PLT in patients with metastatic pancreatic adenocarcinoma (stage IV).

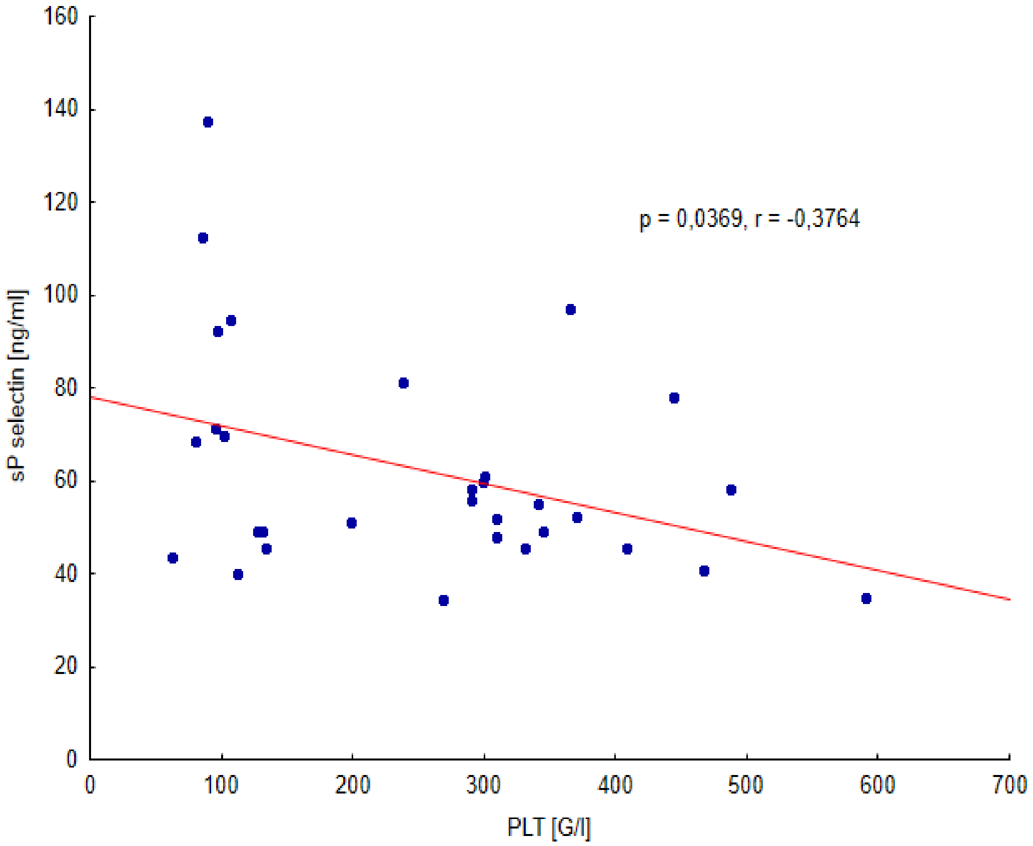

Supplement: Supplementary file 1 [file medicina-56-00677-s001.pdf]
